# Pregnancy and immune thrombocytopenia: New trends

**DOI:** 10.4274/jtgga.galenos.2019.2019.0142

**Published:** 2020-06-08

**Authors:** İrfan Yavaşoğlu, Atakan Turgutkaya

**Affiliations:** 1 Department of Hematology, Adnan Menderes University Medical Faculty, Aydın, Turkey

To the Editor,

I’ve read the article written by Kalaycı et al. (1) with interest. I’d like to emphasize a few points. The term “idiopathic thrombocytopenic purpura” has been abandoned and replaced with “immune thrombocytopenia” following the Vicenza Concensus of 2009. This was as a result of new understanding concerning the pathophysiology of the disease, although the abbreviation “Immune thrombocytopenic purpura (ITP)” remained the same. Also from the diagnosis, up to the first three months, between 3-12 months and after 12 months is now defined as acute, persistent and chronic ITP respectively. The term “primary ITP” should be used unless another condition, such as autoimmune disorders, co-exist. To diagnose ITP, the thrombocyte count should be below 100x10^9^/L (2). ITP is diagnosed in between one and 10 of every 10,000 pregnancies and 30% of cases need therapy. If no other hemostatic abnormality exists, thrombocyte count at delivery should be between 75 to 80x10^9^/L and this view is supported by most guidelines. The “safe” platelet level to prevent post-partum bleeding has been suggested to be 50x10^9^/L in one study similar to yours (3). The American Society of Hematology and International Working group guidelines support intravenous immunoglobulin or corticosteroids as the first line treatment, and they seem to be equally potent in enhancing thrombocyte counts. Intravenous immunoglobulin can achieve a fast but temporary increase in trombocyte counts and is a very useful option for conditions such as delivery or bleeding. Both agents can be given in combination refractory to single agents alone (4). Thrombocyte concentrates should not be used unless there’s a potential life threating bleed. Fetal malformation risk restricts the choice for second-line treatment. Azathioprine may be considered to spare steroids. Anti-RhD immunoglobulin, cyclosporine, and rituximab could be good alternatives, as this has been previously reported although they are not routinely used (4). In an experimental study, it was reported that recombinant human thrombopoietin may be a safe and effective option for the treatment of pregnant ITP patients. Recombinant human thrombopoietin (rhTPO) treatment in ITP at pregnancy is reported in murine models. Significant higher platelet counts were noted in rhTPO-treated groups with no teratogenic effects; supporting that it could be a safe and effective option for refractory cases (5). Although all there is much data, the pregnancy-ITP relationship remains to be illuminated with further prospective studies.
