# Supplementary material for: Pregnancy and immune thrombocytopenia: New trends
Source: J Turk Ger Gynecol Assoc. 2020 Jun 8;21(2):136–7. doi: 10.4274/jtgga.galenos.2019.2019.0142 (PMC7294828; doi:10.4274/jtgga.galenos.2019.2019.0142)
Supplement: Related texts

# Reply: Author’s Response

Dear Editor,

Thank you for precious evaluation and suggestions. The terms and abbreviations which we preferred are consistent with the references included in our manuscript. However, this is a kind reminder of the changes in terms, as well as of some innovations in management. We certainly will consider these comments.

Yours sincerely,

**Hakan Kalaycı, Gülşen Doğan Durdağ, Şafak Yılmaz Baran, Seda Yüksel Şimşek, Songül Alemdaroğlu, Serdinç Özdoğan, Esra Bulgan Kılıçdağ**

**Department of Obstetrics and Gynecology, Başkent University Faculty of Medicine, Adana Application and Research Hospital, Adana, Turkey**
